# Non-coding and Coding Transcriptional Profiles Are Significantly Altered in Pediatric Retinoblastoma Tumors

**DOI:** 10.3389/fonc.2019.00221

**Published:** 2019-04-16

**Authors:** Swetha Rajasekaran, Lakshmi Dhevi Nagarajha Selvan, Kathleen Dotts, Ranjith Kumar, Pukhraj Rishi, Vikas Khetan, Madhoolika Bisht, Karthikeyan Sivaraman, Subrmanian Krishnakumar, Debashis Sahoo, Moray J. Campbell, Sailaja V. Elchuri, Wayne O. Miles

**Affiliations:** ^1^Department of Molecular Genetics, The Ohio State University, Columbus, OH, United States; ^2^The Ohio State University Comprehensive Cancer Center, Columbus, OH,, United States; ^3^Center for RNA Biology, The Ohio State University, Columbus, OH, United States; ^4^Department of Nanobiotechnology, Vision Research Foundation, Sankara Nethralaya, Chennai, India; ^5^Shri Bhagwan Mahavir Vitreoretinal Services and Ocular Oncology Services, Medical Research Foundation, Sankara Nethralaya, Chennai, India; ^6^MedGenome, Bangalore, India; ^7^L&T Department of Ocular Pathology, Vision Research Foundation, Chennai, India; ^8^Department of Pediatrics and Department of Computer Science and Engineering, University of California, San Diego, San Diego, CA, United States; ^9^Division of Pharmaceutics and Pharmaceutical Chemistry, College of Pharmacy, The Ohio State University, Columbus, OH, United States

**Keywords:** retinoblastoma, LNC-RNAs, RNA-sequencing, LNC targeting, DRAIC

## Abstract

Retinoblastoma is a rare pediatric tumor of the retina, caused by the homozygous loss of the Retinoblastoma 1 (RB1) tumor suppressor gene. Previous microarray studies have identified changes in the expression profiles of coding genes; however, our understanding of how non-coding genes change in this tumor is absent. This is an important area of research, as in many adult malignancies, non-coding genes including LNC-RNAs are used as biomarkers to predict outcome and/or relapse. To establish a complete and in-depth RNA profile, of both coding and non-coding genes, in Retinoblastoma tumors, we conducted RNA-seq from a cohort of tumors and normal retina controls. This analysis identified widespread transcriptional changes in the levels of both coding and non-coding genes. Unexpectedly, we also found rare RNA fusion products resulting from genomic alterations, specific to Retinoblastoma tumor samples. We then determined whether these gene expression changes, of both coding and non-coding genes, were also found in a completely independent Retinoblastoma cohort. Using our dataset, we then profiled the potential effects of deregulated LNC-RNAs on the expression of neighboring genes, the entire genome, and on mRNAs that contain a putative area of homology. This analysis showed that most deregulated LNC-RNAs do not act locally to change the transcriptional environment, but potentially function to modulate genes at distant sites. From this analysis, we selected a strongly down-regulated LNC-RNA in Retinoblastoma, DRAIC, and found that restoring DRAIC RNA levels significantly slowed the growth of the Y79 Retinoblastoma cell line. Collectively, our work has generated the first non-coding RNA profile of Retinoblastoma tumors and has found that these tumors show widespread transcriptional deregulation.

## Introduction

The protein produced from the Retinoblastoma 1 (RB1) tumor suppressor gene, pRB (1), functions as a molecular scaffold, to bind to and repress the activator E2 promoter binding factor (E2F) transcription factors 1-3 (E2F1-3) (2). The activator E2F's bind to the promoters of genes necessary for cell cycle progression (3) and apoptosis and induce their expression (2). In this context, pRB plays a central role in controlling the cell cycle and acts a critical check-point to prevent cells with DNA damage or other abnormal features from continuing to divide (4). This activity has led to the widespread inactivation of the pRB/E2F in cancer cells, and a number of genomic events disrupt pRB function (1, 5) or lead to constitutive phosphorylation, and inactivation pRB (6–10). Loss of pRB-activity occurs in the vast majority of tumor types, however background mutations, and the very high frequency of pRB and p53 co-inactivation has made understanding the transcriptional changes solely driven by pRB-loss in tumors difficult to determine.

To identify transcriptional changes to both messenger RNA (mRNA) and long non-coding RNAs (LNC-RNAs) dependent on pRB-loss, we used RNA-sequencing (RNA-seq) to profile Retinoblastoma tumors and normal retinal tissue from patients. Retinoblastoma is a rare pediatric tumor of the Retina that affects around 6,000–8,000 children every year (11, 12). There is currently no targeted therapy for Retinoblastoma (13–16) and many patients suffer secondary tumors (17, 18), or developmental defects, due to treatment. In over 95% of cases, the development of this tumor is driven the homozygous deletion of the Retinoblastoma 1 (RB1) tumor suppressor gene, on chromosome 13p14 (1). The remaining ~5% of Retinoblastoma's are caused by the amplification of the V-Myc Avian Myelocytomatosis Viral Oncogene Neuroblastoma Derived Homolog (N-MYC) gene (19, 20). Loss of TP53 or the upregulation of negative regulators of p53, including MDMX, is also common in Retinoblastoma (21).

As these tumors are collected from pediatric patients, they contain very few genomic alterations and represent an ideal resource for understanding the coding and non-coding transcriptional effects driven by pRB-loss. Determining how loss of pRB changes cells is important for expanding our treatment options for patients with Retinoblastomas, and may provide insights into how RB1 contributes to additional tumor types (22). To expand on previous microarray studies (14, 23–25), we conducted the first in-depth RNA-sequencing (RNA-seq) analysis of Retinoblastoma. This analysis builds upon the mRNA profiling of earlier studies and expands the coverage of non-coding changes.

## Results

### RNA-Sequencing From Retinoblastoma and Retinal Tissue

**Figure 1:**
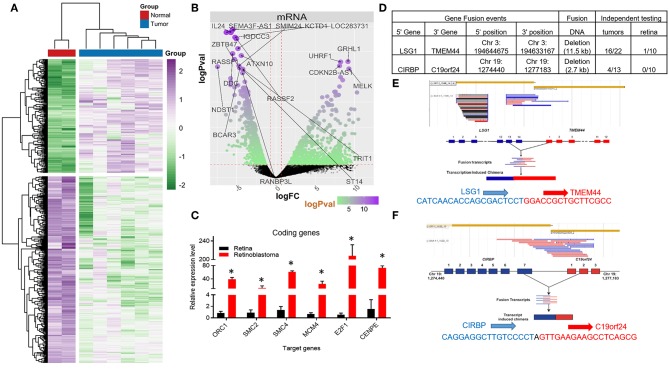
Coding RNA changes and RNA fusion products in Retinoblastoma tumors. **(A)** Heat map of transcriptional changes in Retinoblastoma tumors and normal retinal samples. **(B)** Volcano plot of the log fold change (logFC) (x-axis) and the log *p*-value (logPval) (y-axis) of the coding (mRNA) RNA changes in tumors and normal tissue. **(C)** RT-PCR Relative expression of Six E2F targets genes (ORC1, SMC2, SMC4, MCM4, E2F1, and CENPE) from four control retinal tissues and three Retinoblastoma tumors from an independent cohort (^*^p < 0.05). **(D)** Table detailing the RNA fusion products detected in the RNA-seq data, including independent testing in Retinoblastoma primary tumors and control retinal samples. **(E)** Screenshot and sequencing data of the LSG1 and TMEM44 fusion RNA detected in Retinoblastoma tumors. **(F)** Screenshot and sequencing data of the CIRBP and C19orf24 fusion RNA detected in Retinoblastoma tumors.

To determine how the loss of the Retinoblastoma 1 (RB1) tumor-suppressor gene changes the expression of coding and non-coding RNAs in tumors, we conducted RNA-sequencing (RNA-seq) from 7 Retinoblastoma patients. The tumors were removed from patients between 8 months and 6 years of age, with a mean age of 2 years and 8 months. Importantly, the gender demographics of these patients is equally split between males (4) and females (3), and between invasive (3), and non-invasive (4) tumors. A complete list of the patient and control sample demographics is included in Table 1. We also conducted RNA-seq on control Retinal samples and incorporated additional public retinal datasets to build a transcriptional profile of normal retina (Supplementary Tables 1, 2). The RNA-seq profiles of these samples were then separated, and clustered to measure transcriptional changes in Retinoblastoma tumors (Figure 1A). Using normalized values, we found significant and recurrent changes in both coding and long non-coding RNAs (LNC-RNAs) within the tumors (Figure 1B).

**Table 1:** Clinical characteristics of the patients and tissue used in the RNA-Seq analysis.

**Patient ID**	**RNASeq_Ref ID**	**Pathology Ref ID**	**Age at the time of enucleation**	**Histo pathology report**	**Sex**	**Unilateral/Bilateral**	**Chemo status before enucleation**	**Invasiveness**
C1	48428	12 years Retina	12 Y	N/A	Unknown	N/A	N/A	N/A
1	48429	67/13	6 Y	Retinoblastoma, poorly differentiated, focal invasion of choroid measuring more than 3mm. There is prelaminar and laminar invasion of the optic nerve present. Hyperchromatic cells are seen in the trabecular meshwork and over the iris surface. The post laminar portion and the surgical end of optic nerve are free from tumor cells.	Male	OD (IIRC Grade E)	No	Invasive
2	48430	1563/15	3 Y	Retinoblastoma, poorly differentiated, endophytic growth pattern. No invasion of choroid. No invasion of optic nerve.	Male	OS (IIRC Grade E)	No	Non-Invasive
C2	48431	5 years Retina	5 Y	N/A	Unknown	N/A	N/A	N/A
3	48432	1561-15	2 Y	Retinoblastoma well differentiated. No invasion of iris.No invasion of choroid and optic nerve.	Female	OD (IIRC Grade E)	No	Non-Invasive
4	48433	1630-15	3 Y	Retinoblastoma, poorly differentiated. No invasion of choroid. No invasion of optic nerve.	Female	OD (IIRC Grade D)	No	Non-Invasive
5	48434	647-13	1 Y	Retinoblastoma, well differentiated, focal retinal pigment epithelial invasion, there is no invasion of choroid. Preliminar invasion of optic nerve is seen. The laminar, post laminar and surgical end of optic nerve is free from tumor.	Female	OD	Yes (Two cycles)	Invasive
6	48435	1548-14	3 Y	Retinoblastoma poorly differentiated.	Male	OS (IIRC Grade D)	No	Non-Invasive
				There is no invasion of choroid. There is No invasion of optic nerve. No invasion into iris stroma.				
C3	48436	22 years Retina	22 Y	N/A	Unknown	N/A	N/A	N/A
7	48437	768-13	8 Months	Retinoblastoma, well differentiated, no invasion of choroid. There is prelaminar, laminar, post laminar invasion of optic nerve. Separate section at the surgical end of optic nerve is free from tumor cells. There is no scleral invasion.	Male	OD (IIRC Grade E)	No	Invasive

We then used Gene Ontology (GO) analysis (26, 27) to identify biological processes altered in Retinoblastoma tumors. In accordance with previous microarray studies, we found DNA replication and mitotic progression genes were strongly upregulated and retina specific genes downregulated in tumors (Supplementary Figures 1A–C). In addition, the majority of upregulated genes (>3-fold) were either E2F (28, 29) or MYC target genes (Supplementary Figures 1D,E) (30). To independently test the levels of coding gene expression, we used RT-PCR to measure the levels of a subset of E2F-target genes from an independent cohort of tumors and retina controls (Supplementary Figure 2). This analysis confirmed that E2F target genes are consistently increased in RB1-mutant Retinoblastomas (Figure 1C). We next compared our Retinoblastoma transcriptional profile to datasets from other pediactric tumors. From this analysis, we found very limited overlap suggesting the gene signature of Retinoblastoma is unique (Supplementary Figure 3A).

### Aberrant RNA Processing Events in Retinoblastoma

Previous microarray studies of Retinoblastoma tumors have provided important insights into the coding RNA changes in these tumors. However, these technologies are limited in their capacity to detect RNA fusion products. To address this, we searched for RNA-fusions within our RNA-seq dataset and identified 22 putative RNA fusion events. From this collection, we selected two putative RNA hybrids for detailed examination, using RT-PCR. For this RT-PCR based evaluation, we used 10 normal retinal tissue samples and a larger cohort (>13) of Retinoblastoma tumors (Figure 1D). From this work, we were able to detect RNA from the LSG1-TMEM44 RNA fusion in 73% of tumors, whilst only 1 control sample had detectable levels (Figures 1D,E, Supplementary Figure 3B). In contrast, the CIRBP-C19orf24 fusion occurred in 31% of tumors but was not found in control samples (Figures 1D,F, Supplementary Figure 3B). We then sequenced these RT-PCR products and confirmed the RNA fusion (Figures 1E,F). From this analysis, we have identified and confirmed the presence of RNA-fusions in Retinoblastoma.

### Non-coding RNA Changes in Tumors

**Figure 2:**
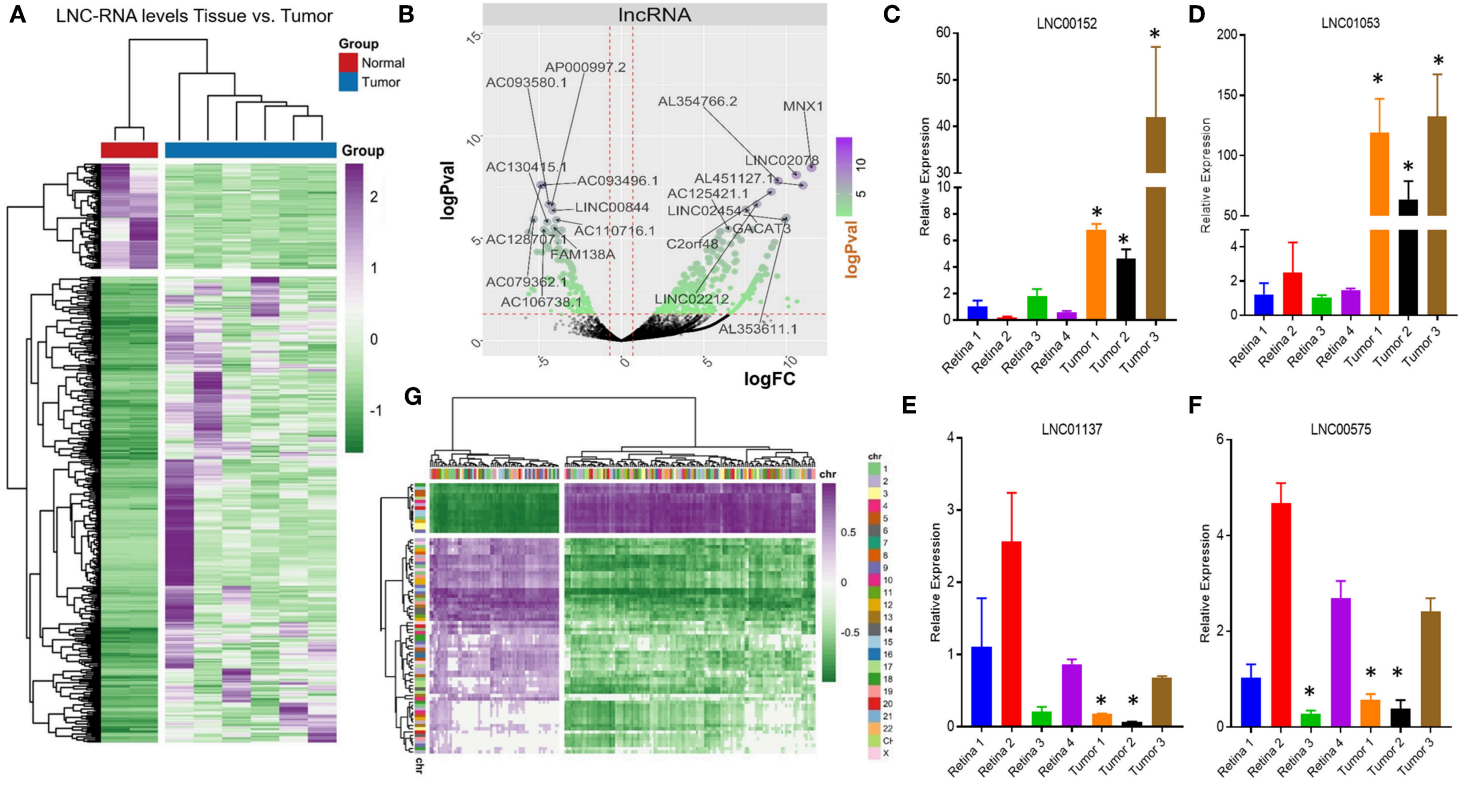
Non-coding RNA expression changes in Retinoblastoma tumors. **(A)** Heat map of non-coding transcriptional changes in Retinoblastoma tumors and normal retina samples. **(B)** Volcano plot of the log fold change (logFC) (x-axis) and the log *p*-value (logPval) (y-axis) of the non-coding RNA changes in tumors and normal tissue. **(C)** RT-PCR Relative expression of LNC00152 from four control retinal tissues and three Retinoblastoma tumors from an independent cohort (^*^p < 0.05). **(D)** RT-PCR Relative expression of LNC01053 from four control retinal tissues and three Retinoblastoma tumors from an independent cohort (^*^p < 0.05). **(E)** RT-PCR Relative expression of LNC01137 from four control retinal tissues and three Retinoblastoma tumors from an independent cohort (^*^p < 0.05). **(F)** RT-PCR Relative expression of LNC00575 from four control retinal tissues and three Retinoblastoma tumors from an independent cohort (^*^p < 0.05). **(G)** Heat map of non-coding transcriptional changes from Retinoblastoma tumors, compared to anti-correlated deregulated mRNA clustered by genomic location.

Very little is known about how loss of the pRB tumor-suppressor gene affects the transcription of LNC-RNAs in tumors. To address this, we next analyzed the expression patterns of LNC-RNAs in Retinoblastoma and found widespread alterations in LNC-RNA levels (Figure 2A). Subsets of LNC-RNAs were both up and downregulated in tumors and could be used to sub-classify tumors from normal tissue (Figure 2A). This analysis identified groups of LNC-RNAs that show significant alterations in their levels (Figure 2B). As many of these genes are poorly characterized, we chose to measure the levels of a subset of these de-regulated LNC-RNAs in an independent tumor and control cohort. We measured each candidate LNC-RNA using RT-PCR and found that our results correlated well with our RNA-seq analysis. Up-regulated LNC-RNAs (LNC00152 and LNC01053) were consistently significantly increased (Figures 2C,D), whilst down-regulated LNC-RNAs (LNC01137 and LNC00575) were decreased in 2 of the 3 tumors (Figures 2E,F). Predicting how LNC-RNAs may function in tumors is challenging and is dependent on a number of factors. We next investigated how the expression of each deregulated LNC-RNA correlated with the expression of genes in the entire genome, locally, or *in trans*. We first compared the genomic loci of significantly up or downregulated LNC-RNAs, with the expression of deregulated coding genes that displayed reciprocal patterns. From this assay, we found that de-regulated LNC-RNAs (either up or downregulated) showed statistically significant anti-correlates with coding genes, however, these patterns were not restricted by chromosomal location (Figure 2G). This analysis suggested that the majority of transcriptionally altered LNC-RNAs may not affect the local expression of genes.

**Figure 3:**
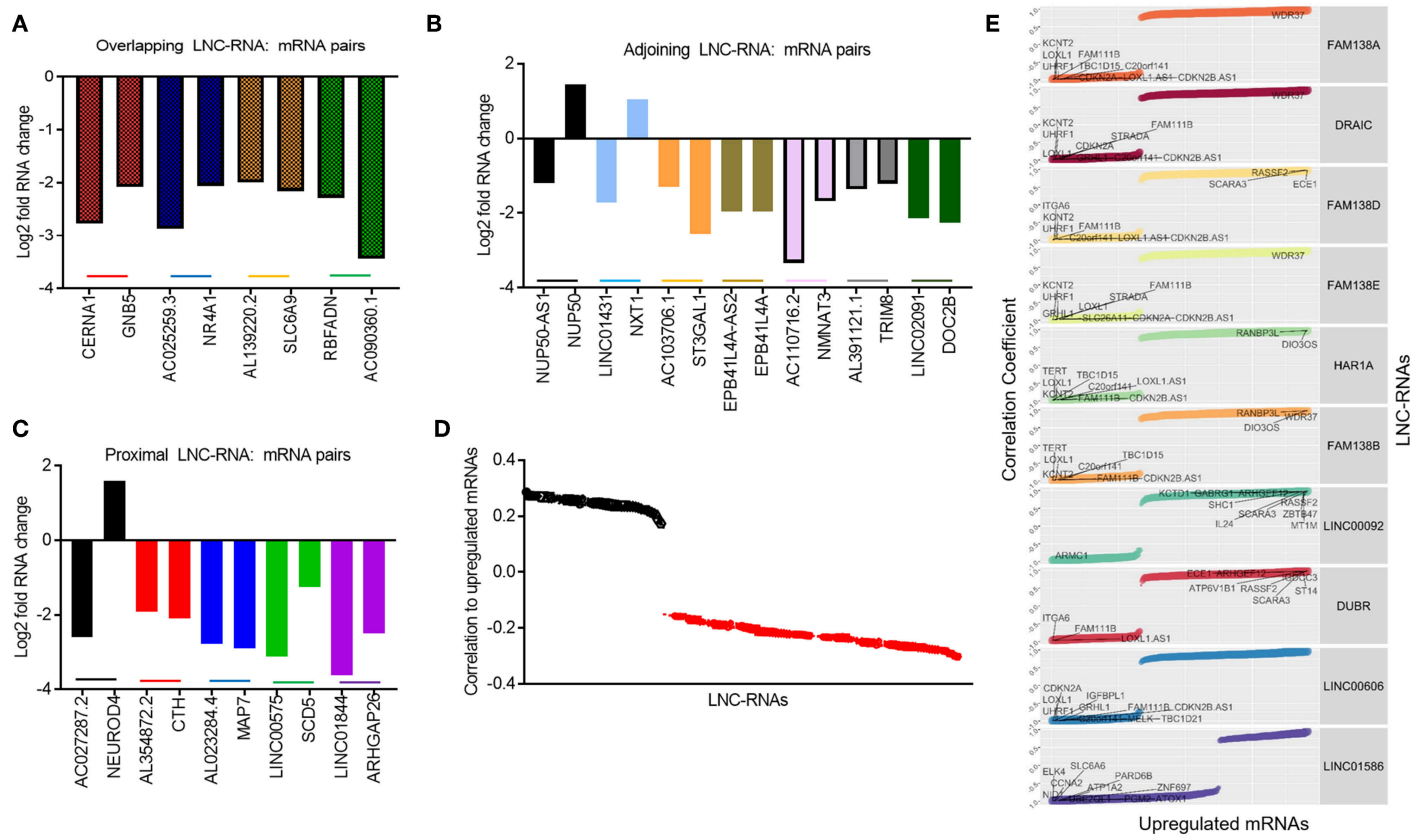
Predicting local and distant potential LNC-RNA: RNA regulation. **(A)** Graph of significantly changed LNC-RNA: mRNA pairs from overlapping loci. **(B)** Graph of significantly changed LNC-RNA: mRNA pairs from adjoining loci. **(C)** Graph of significantly changed LNC-RNA: mRNA pairs from proximal loci. **(D)** Average correlations of deregulated LNC-RNAs to mRNA changes in Retinoblastoma tumors (Black dots: Average positive correlation, Red dots: Average negative correlation). **(E)** Examples of deregulated LNC-RNAs (FAM138A, DRAIC, FAM138D, FAM138E, HAR1A, FAM138B, LNC00092, DUBR, LNC00606, and LNC01586) and relative correlation coefficient to coding gene changes.

Despite this global pattern, we did identify a subset of LNC-RNAs, whose changes in expression correlated with that of their neighboring gene. In particular, reduced expression of the LNC-RNAs: CERNA1, AC025259.3, AL139220.2, and AC090360.1, all correlated with lower mRNA levels of the genes that they overlap with, including: GNB5, NR4A1, SLC6A9, and RBFADN (Figure 3A). Interestingly, when we measured adjoining LNC-RNA: mRNA pairs, we observed two different patterns. In the cases of NUP50-AS1 and LNC01431, we found that LNC-RNA downregulation was associated with increased expression of their neighboring genes, NUP50 and NXT1 (Figure 3B, Black and Blue bars). More commonly however, we found that reduced LNC-RNA levels, AC103706.1, EPB41L4A-AS2, AC110716.2, AL391121.1, and LNC02091, was correlated with lower mRNA levels of their adjoining genes, namely: ST3GAL1, EPB41L4A, NMNAT3, TRIM8, and DOC2B (Figure 3B). Analysis of the correlation in expression of proximal LNC-RNAs and their neighboring genes identified both anti (AC027287.2-NEUROD4) and positively correlated associations (AL354872.2-CTH, AL023284.4-MAP7, LNC00575-SCD5, and LNC01844-ARHGAP26) (Figure 3C). Collectively, this analysis identified a subset of LNC-RNAs and their neighboring genes that have altered expression patterns in Retinoblastoma.

The majority of LNC-RNA expression changes in these tumors do not correlate with genes geographically associated with them. We next examined whether LNC-RNAs with altered mRNA levels correlate with the expression any other genes across the genome. To do this, we stratified the LNC-RNAs and measured the average correlation of changed mRNAs. From this analysis, we identified several LNC-RNAs that show both average positive and negative relationships with the altered mRNAs (Figure 3D). We next selected 10 of these LNC-RNAs for closer examination and found that each LNC-RNA was significantly correlated to many deregulated genes in Retinoblastoma (Figure 3E). Collectively, this analysis shows that LNC-RNAs have diverse and putatively complex relationships with the expression of their neighboring genes and genes throughout the genome.

### Non-coding RNA Changes and Their Putative Effects *in trans*

We next investigated the putative interaction between deregulated LNC-RNAs in Retinoblastoma and mRNAs that contain regions that could be putatively bound by the LNC-RNA. To evaluate this, we identified gene expression changes in coding genes that had highly homologous regions to those of de-regulated LNC-RNAs. We then computationally measured the potential for RNA-RNA duplexes to form, between the coding gene, and the LNC-RNA (31). Our analysis used the RNA-RNA interaction database (31).

**Figure 4:**
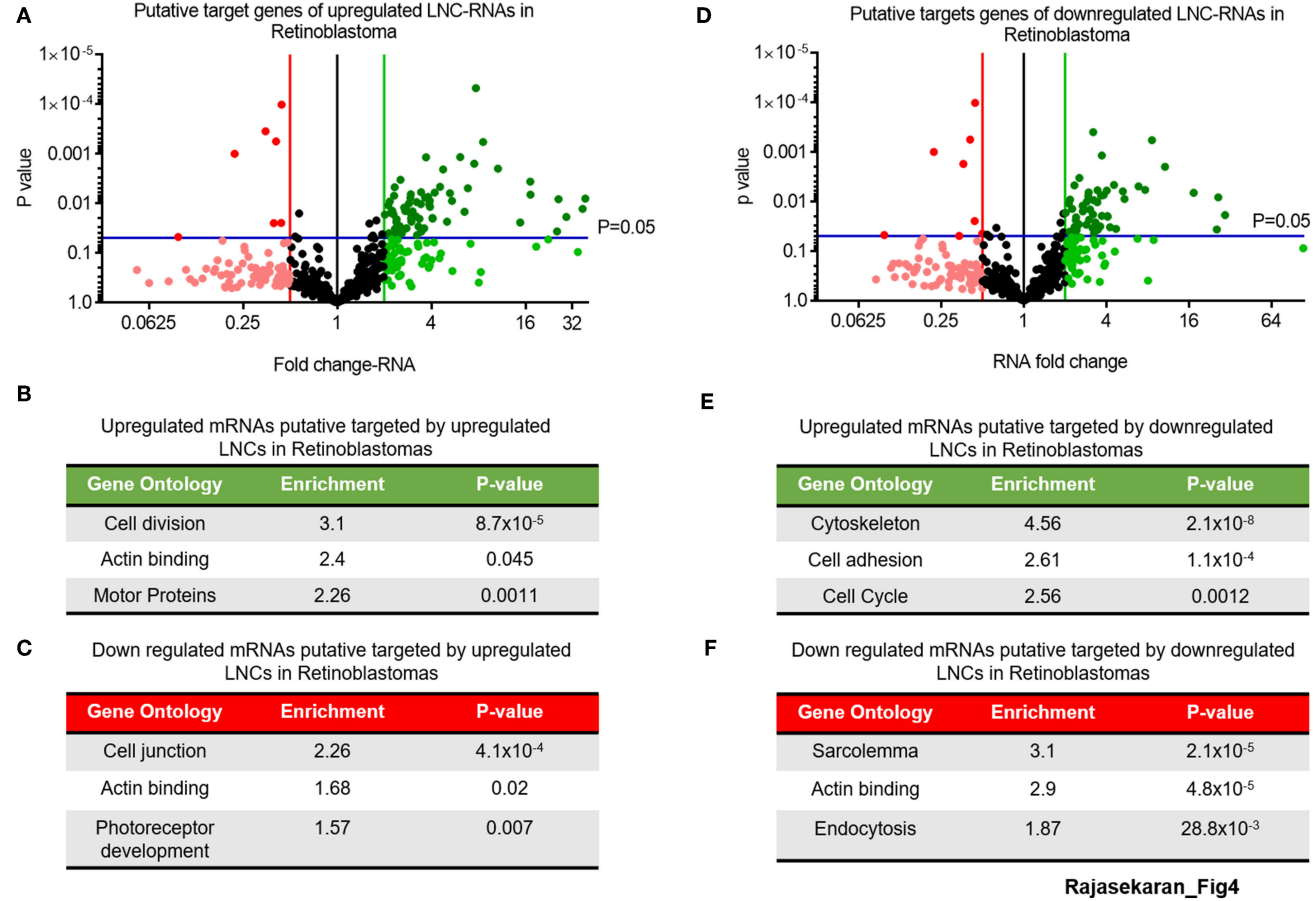
Putative Molecular processes regulated *in-trans* by significantly changed LNC-RNAs in Retinoblastoma. **(A)** Volcano plot of expression of mRNAs targeted by upregulated LNC-RNAs in Retinoblastoma tumors. Green line represents 2-fold increase, Red line represents 2-fold decrease, Blue lines represent statistically significant cutoff of *p*-value of 0.05. Deep Green circles: *p* < 0.05 and >2-fold increased, Light Green circles: *p* > 0.05 and >2-fold increased, Black Circles: unchanged, Light Red circles: *p* > 0.05 and >2-fold decreased and Deep Red circles: *p* < 0.05 and >2-fold decreased. **(B)** Gene Ontology of the mRNAs upregulated and putatively targeted by upregulated LNC-RNAs. **(C)** Gene Ontology of the mRNAs downregulated and putatively targeted by upregulated LNC-RNAs. **(D)** Volcano plot of expression of mRNAs targeted by downregulated LNC-RNAs in Retinoblastoma tumors. Green line represents 2-fold increase, Red line represents 2-fold decrease, Blue lines represent statistically significant cutoff of *p*-value of 0.05. Deep Green circles: *p* < 0.05 and >2-fold increased, Light Green circles: *p* > 0.05 and >2-fold increased, Black Circles: unchanged, Light Red circles: *p* > 0.05 and >2-fold decreased and Deep Red circles: *p* < 0.05 and >2-fold decreased. **(E)** Gene Ontology of the mRNAs upregulated and putatively targeted by downregulated LNC-RNAs. **(F)** Gene Ontology of the mRNAs downregulated and putatively targeted by downregulated LNC-RNAs.

This analysis of the putative targets of upregulated LNC-RNAs in Retinoblastoma tumors identified a group of 68 genes that are increased and 6 genes that are decreased (2-fold changed and *p* > 0.05) (Figure 4A, UP: Dark green balls, DOWN: Bright red balls). Gene Ontology of these increased mRNAs (>2-fold), that are putatively targeted by upregulated LNC-RNAs, showed that they function in processes supporting cell division and microtubule function (Figure 4B). In the genes downregulated (2-fold lower), we identified enrichments in genes modulating cellular adhesion and retinal development (Figure 4C). We next determined the putative targets for the LNC-RNAs downregulated in Retinoblastoma tumors, using the same stringency parameters. From this, we found 54 putative mRNAs that are increased and 8 genes with decreased expression (2-fold changed and *p* > 0.05) (Figure 4D, UP: Dark green balls, DOWN: Bright red balls). Gene Ontology analysis of these increased mRNAs, that are putatively targeted by down-regulated LNC-RNAs, showed that cell attachments and proliferation factors are the molecular processes putatively changed by lower LNC-RNA levels (Figure 4E). Putative LNC-RNA targets with lower expression levels in Retinoblastoma tumors are involved in muscle associated processes (Sarcolemma) and Endocytosis (Figure 4F). Collectively, this analysis highlights the potential complexity of LNC-RNA function in Retinoblastoma.

**Figure 5:**
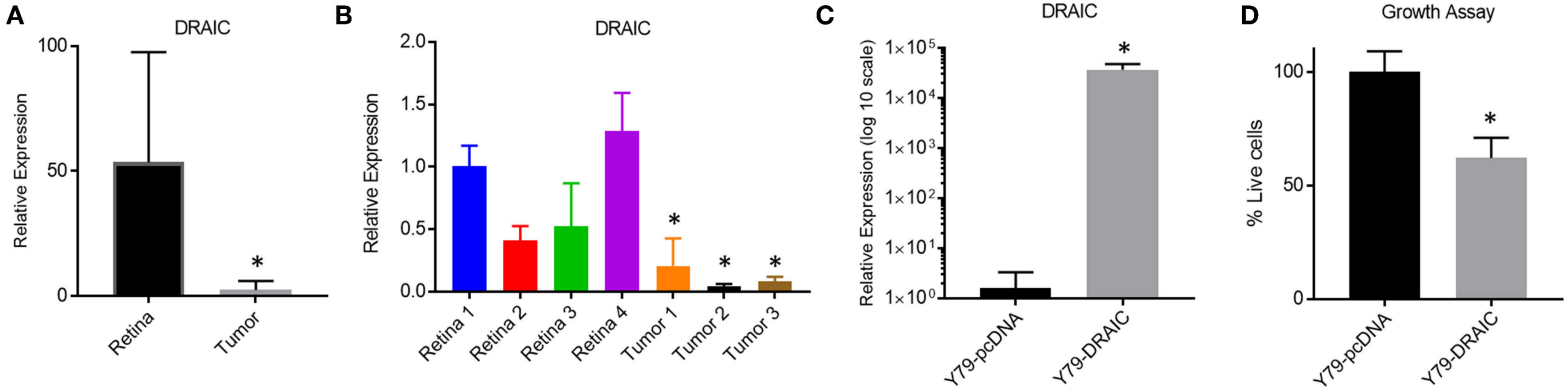
Re-Expression of the DRAIC LNC-RNA slows the growth of Retinoblastoma cell lines. **(A)** Expression levels of DRAIC LNC-RNA in normal Retina vs. Retinoblastoma tumors (*p* < 0.05). **(B)** RT-PCR of DRAIC levels in an independent tumor cohort (*p* < 0.05). **(C)** RT-PCR of Y79 cells expressing either pcDNA-DRAIC or an empty pcDNA control. **(D)** Growth assay of Y79 cells transfected with either pcDNA-DRAIC or an empty pcDNA control.

We next investigated how modulating the levels of a down-regulated LNC-RNA in Retinoblastoma affected the growth of Y79 Retinoblastoma cells. For this, we selected the DRAIC LNC-RNA, as DRAIC levels are strongly decreased in tumors (Figure 5A) and in our independent Retinoblastoma cohort (Figure 5B). We next tested how DRAIC over-expression contributed to the growth of Retinoblastoma cells. To do this, we transfected cells with either empty pCDNA plasmids or pcDNA-DRAIC over-expression plasmids (Figure 5C) (32) and measured Y79 growth. From these experiments, we found that DRAIC over-expression significantly slows Y79 growth (Figure 5D), without promoting cell death.

## Discussion

Inactivation of the RB/E2F pathway is a common feature of cancer (22). In this analysis, we sought to understand how the homozygous deletion of the RB1 gene changed the transcriptional profile of both coding and non-coding genes in the rare pediatric tumor, Retinoblastoma. To do this, we compared the gene expression of Retinoblastoma tumors with that from normal retinal tissue using RNA-sequencing. This represents the first time that such an approach has been used to generate a complete transcriptional profile of these tumors and builds upon previous coding RNA studies (23–25, 33). Our work expands our coverage of the genome for Retinoblastoma and includes the expression from non-coding regions of the genome, including long non-coding RNAs (LNC-RNAs), anti-sense RNAs, and transposable elements. This new in-depth analysis of RNA changes, coupled with our recent proteomic profiling of this cohort of Retinoblastoma (34), provides a new resource for the Retinoblastoma and RB-loss research community.

In agreement with previous microarray based studies (23–25, 33), we find very strong transcriptional upregulation of E2F target genes involved in cell cycle progression and apoptosis. In contrast, p53 and MYC target genes are only mildly affected. Most of the down regulated genes are associated with retina cell differentiation and ocular development. These results are consistent with retinal cells when they abnormally differentiate and cause lineage specific gene expression changes.

As RNA-seq provides a unique platform for detecting rare fusion or trans-spliced transcripts, we investigated the frequency of these events in Retinoblastoma. There are multiple potential mechanisms for generating the hybrid RNAs, including chromosomal translocation, deletions or inversions, gene fusion, or aberrant splicing events. We independently evaluated each dataset to identify potential fusion transcripts, as these have been shown to have potential oncogenic (35) or predictive roles (36) in different malignancies. Our analysis revealed a small subset of putative fusion transcripts in our tumor cohort between consecutive genes, where transcripts of the two genes are transcribed into one chimeric transcript by splicing out the intergenic region. Further independent testing of these candidate genes, using RT-PCR, found 2 *bone fide* fusion transcripts that occurred at high frequencies in Retinoblastoma tumors, relative to normal retinal tissue. Although much more work is needed to test the possible function of these RNAs, this work suggests that RNA-fusion products are common in Retinoblastoma tumors.

This work represents the first genome-wide profiles of non-coding RNA changes in Retinoblastoma tumors. Our profiles identified significant and widespread differences in the levels of anti-sense, LNC-RNAs and transposable elements in tumor samples, compared to normal retinal tissue. Although there is some inter-tumoral variation between LNC-RNA levels, the majority show consistent changes which could be independently confirmed in an unrelated Retinoblastoma cohort. Determining the potential roles of LNC-RNAs can be challenging, as they can potentially function via many mechanisms to affect local or distant gene expression or directly interact with other RNAs to alter their stability and/or translation capacity. To address this complex question, we conducted an exhaustive analysis of how altered LNC-RNAs may contribute to the relative mRNA levels in Retinoblastoma.

First, we investigated how each altered LNC-RNA correlated with deregulated mRNA levels, based on chromosome location. These predictions suggest that although each LNC-RNA did show anti-correlates with several mRNAs, these were spread throughout the genome, suggesting that most of the changed LNC-RNAs in Retinoblastoma do not function *in cis*. In agreement with this hypothesis, we detected only 16 LNC-RNAs that showed tight expression correlations with their overlapping, adjoining or proximal gene partners. Second, by generating correlation efficiencies for each of the 400 most deregulated LNC-RNAs in our tumors to the mRNA changes, we found that many LNC-RNAs showed a relative positive or negative relationship to mRNA levels. Third, using new LNC-RNA: mRNA interaction platforms followed by gene ontology analysis of these putative interactions, we found that many up and down regulated LNC-RNAs have strong potential interactions with genes involved in cellular processes that sustain Retinoblastoma growth including cell division and cell attachment. These data provide a complete analysis of the transcriptional changes for both coding and non-coding genes in the pediatric Retinoblastoma tumor.

To measure how altered LNC-RNAs may contribute to Retinoblastoma growth, we evaluated how re-expressing a down-regulated LNC-RNA in Y79 Retinoblastoma cell lines altered cell number. DRAIC expression slowed Y79 cell growth compared to controls, in support of previous data from lung cancer, showing that reducing the expression of DRAIC back to physiological levels changes the proliferation potential of cells. These experiments show that modulating the levels the DRAIC LNC-RNA does affect Retinoblastoma cells.

## Methods

### Patient Samples for RNA-seq

The 2 normal Retinas were obtained from donor eye balls from the PU Shah eye bank. The age was 12 and 22 years, respectively. The Retinoblastoma tumors were obtained from informed consent provided by the parents of the children undergoing treatment at Sankara Nethralaya, India. The protocol was approved by institutional review board on Ethical practices for research at Sankara Nethralaya. The pathological characterizations of the tumors are presented in Table 2. For RNA-sequencing studies, the tumors and retinas were stored at −80°C until further use. The medical record pathology numbers of these samples were de-identified before the tumors were released by the hospital. Each sample was then assigned an identification number by the hospital. The RNA was then used for RNA-sequencing. The samples were named from 48,428 to 48,437.

**Table 2:** Primers.

	**Target**	**Forward primer**	**Reverse primer**
1	ORC1	CCCTATCAGTGGGGGACAGA	ATGGGGAGTAGAGGTCGCTT
2	SMC2	TCAGCCAGATGTATTGCACCA	CACATGAACGTTGTCAGGGC
3	SMC4	CCTGTTGTCATGCACTGGACT	TCGGTCATCTTTTTCGCCCA
4	TOP2A	AGCTGGATCAGTGGCTGAAAT	GCCTGGTACCAAACTGACCA
5	MCM4	TGTTTGCTCACAATGATCTCG	CGAATAGGCACAGCTCGATA
6	E2F1	ACTCCTCGCAGATCGTCATCATCT	GGACGTTGGTGATGTCATAGATGCG
7	CENPE	GATTTGGATGAATTTGAGGCTCT	ACTTCTGCATGCTTAACTAAATTCT
8	LNC00505	GTGAAGACCCCCTTTCCCAC	AGATGCTGGCTAGTTTGGGG
9	LNC01053	CTGTCCCTATCTGGAGCCCT	GAAAGATGTGTGCGTGACCG
10	LNC00152	CCAGCACCTCTACCTGTTGC	GCCAGACAAATGGGAAACCG
11	LNC01155	ACCTTCTTGGCCCTGCTTAG	TAGAGGGTGGCCCTTAGTGA
12	LNC-PINT	CGCAGAGGGACAAATCCAGT	CCCCGGAGAGCAATGAGTTT
13	LNC00844	TCTGATAGGAGGATGGGGGTG	CTTAGCCATGCAAGAAACCTCC
14	LNC01137	AGCGATCTTGGGGGAAGTTG	GGGTTAGGGAGTGGCATCAC
15	LNC00575	GAATTGGCACAGATCCAGAGC	GGTTCTCATCCCCTGAAGTCT
16	LNC00606	CACTGCTTTGGTCAGGGAGT	ACTCTCCTTGTCAGCGGTTG
17	β-actin	TCACCCACACTGTGCCCATCTACG	CAGCGGAACCGCTCATTGCCAATG
18	LSG1_TMEM44	AAGCTGCTGTACTGCCATCCTC	CAGGAGGAGGCGCAGATC
19	CIRBP_C19orf24	AGGACTCGGGGAAGGGTG	ATTCCGGGACTCAAACACCG
20	DRAIC	TGAACTCAACTCCTGAGAAGGAC	CGCTCTCAGACTCTTCAGTTCTC
21	β-actin	TCACCCACACTGTGCCCATCTACG	CAGCGGAACCGCTCATTGCCAATG

### RNA Extraction for RNA-seq

The Retinae and tumors were thawed, and RNA was extracted using TRIzol reagent (#15596026, Thermo Fisher Scientific) according to the manufacturer's instructions. The samples were homogenized in TRIzol reagent and incubated at room temperature for 5 min. Next, chloroform was added, and the tubes were mixed vigorously for 15 s followed by incubation at room temperature for 2–3 min. Next, the samples were centrifuged at 4°C for 15 min at 12,000 g. RNA, which is part of the aqueous phase, is collected cautiously and is precipitated using isopropanol, followed by 75% ethanol washes. After the washes, RNA pellet is dried completely (5–10 min) before re-suspending in nuclease free water. Total RNA thus extracted is qualified on 2,200 Tape Station using the RNA specific Tapes and reagents (#5067-5576, 5577, Agilent Technologies). Quantification of the RNA was done using Qubit 3.0 Fluorimeter (#Q33216, Thermo Fisher Scientific) with an RNA HS Assay (#Q32852, Thermo Fisher Scientific).

### RNA Library Preparation and RNA-seq

Once the RNA passed QC, it was taken up for library preparation using the TruSeq RNA Library Prep Kit V2 (#RS-122-2001/RS-122-2002, Illumina Inc.). During the library preparation, the Ribosomal RNA (rRNA) were removed using biotinylated, rRNA specific oligo's combined with Ribo-zero rRNA removal beads (#MRZG12324, Illumina Inc.). Following purification, the Ribo-depleted RNA is subjected to fragmentation in the presence of divalent cations at elevated temperatures. The cleaved RNA fragments were copied into first strand cDNA using reverse transcriptase and random primers. Then, second strand cDNA synthesis was performed, using DNA polymerase I and RNase H. These cDNA fragments will go through the process of repairing the ends, addition of a single “A” base, followed by ligation of the adapters. The ligation product was purified using Agencourt AMPure XP beads (#A63882, Beckman Coulter) and enriched using PCR to create the final cDNA library for sequencing. After a final cleanup with Agencourt AMPure XP beads, the library was quantified using Qubit DNA Assay and the fragments were assessed using DNA Tape Station D1000 Screen Tape (#5067-5582,5583, Agilent Technologies). The quantified libraries were then clonally amplified on a cBOT and sequenced on the HiSeq2500 with 100 bp paired end chemistry. 5′AATGATACGGCGACCACCGAGATCTACACTCTTTCCCTACACGACGCTCTTCCGATCT3′

The TruSeq Universal Adapter. The TruSeq Illumina Index Adapter consisted of following sequence: 5′GATCGGAAGAGCACACGTCTGAACTCCAGTCAC[INDEX]ATCTCGTATGCCGTCTTCTGCTTG3′.

### Bioinformatics

The raw fastq files generated in sequencing were first subjected to a quality check to filter out substandard data. During quality control, the following parameters were checked: base quality score distribution, sequence quality score distribution, average base content per read, GC distribution in the reads, PCR amplification issue, check for over-represented sequences and finally, adapter trimming. Based on the QC report, the reads having a phred quality score of Q30 were either removed or trimmed as per requirement. The adapter trimming was done using fastq-mcf v-1.04.676 and cutadapt v-1.8dev (ftp://ftp.ensembl.org/pub/release75/gtf/homo_sapiens/Homo_sapiens.GRCh37). The processed data were then checked for the presence of unwanted sequences or contamination (e.g., non-polyA tailed RNAs, mitochondrial genome sequences, ribosomal RNAs, transfer RNAs, adapter sequences, and others), and those contaminations were removed using Bowtie2 v-2.2.4 (37). The first step of analyzing the processed RNA-seq data is to align them to the reference human genome. To do this, the paired-end reads were aligned to reference human genome, GRCh37/hg19 (2), using HISAT v-0.1.7 (38). The aligned reads were then subjected to expression estimation of individual genes and transcripts by calculating the FPKM values (Fragments Per Kilo base of transcript per Million mapped reads) using cufflinks v-2.2.1 (39). Finally, the differential expressions of genes were carried out using cuffdiff v-2.2.1 & DESEQ2 (40). Genes with a log2 fold change of more than 1 or less than −1 and a *p* < 0.05 were termed as differentially expressed genes.

Global changes in mRNA and biological triplicate samples per experimental condition were analyzed using RNA-Seq, specifically with the consensus Bioconductor (41, 42) workflow; FASTQC for trimming, genomic alignment in Rsubread, and differential expression with limma (43) and visualization (pheatmap) and volcano plots were generated in ggplot2.

Subsequently, the Pearson's correlation was measured for each of the top differentially expressed mRNAs or lncRNAs, and a cross-correlation of q values was generated. These significant correlations, >0.3 or <-0.3, were also visualized in pheatmap with the chromosome and genomic start location used to annotate each species. Finally, the 10 lncRNA with the greatest sum of correlations were selected and the mRNA correlations illustrated with the most significant mRNA species identified.

### LNC-RNA Target Prediction

LNC-RNAs that were statistically significantly up or downregulated in Retinoblastoma tumors, compared to normal retinal tissue, were profiled to determine their putative mRNA targets. LNC-RNAs were retained for this analysis if they had SUMENERGY scores of above 2,000 for at least 10 targets and all putative substrates with SUMENERGY above 1,500 were included. This step was taken to reduce the number of weak substrates included in the Gene Ontology. LNC-RNAs that did not have this criteria were excluded, as were mRNAs that were predicted in >4 LNC-RNAs as this was taken as a false positive. LNC-RNAs were entered into the RNA-RNA interaction database (human) and the predicted targets were extracted. These mRNAs, their associated SUMENERGY score and change in expression, were used to sub-group the putative LNC-RNA targets in the tumor cohort. These groups were then used in Gene Ontology analysis using DAVID (44, 45).

### LNC-RNA Neighboring Gene Analysis

LNC-RNAs are known to regulate gene expression in cis-acting and trans-acting ways. We carried out neighboring gene analysis and LNC RNA-mRNA interaction analysis to identify the probable targets of LNC-RNA. LNC-RNAs identified to be differentially expressed were classified based on genomic location, and this was done with any other gene (irrespective of coding nature). The following distance parameters were used: Overlap is within any other gene, Adjoining is within 5 kb of another gene, Proximal is within 10 kb of another gene, Others >10 kb of another gene. Those neighboring protein-coding genes that were found to be significantly expressed were considered to be influenced by LNC-RNA.

### Tumors and RNA Extraction Method for RT-PCR

Retinoblastoma samples were provided by the Cooperative Human Tissue Network, a National Cancer Institute supported resource. Other investigators may have received samples from these same tissue specimens. Normal retinal tissue was obtained from warm autopsy samples from Mohamed Abdel-Rahman at the Ohio State University. RNA was extracted from each of the three retinoblastoma tissue samples and four retina tissue samples that were received snap frozen. To each sample, 1 mL TRIzol Reagent (Invitrogen) was added to 10 mg of tissue and the samples were homogenized using a homogenizer and incubated at room temperature for 5 min. 0.2 mL of chloroform was added to the samples and incubated at room temperature for 2 min, then the samples were centrifuged for 15 min at 12,000 × g at 4°C. The upper aqueous phase containing the RNA was transferred to a new tube and 0.5 mL of isopropanol was added, then this mixture was incubated at room temperature for 10 min. The samples were centrifuged for 10 min at 12,000 × g at 4°C and a white pellet formed. The supernatant was discarded and the pellet re-suspended in 1 mL of 75% ethanol. The sample was briefly vortexed and then centrifuged for 5 min at 7,500 × g at 4°C. The supernatant was discarded and the RNA pellet air dried for 10 min. The pellet was re-suspended in 20 μL of RNase-free water and incubated in a 60°C water bath for 15 min. The RNA was then quantified using a BioTek Epoch Microplate Spectrophotometer.

### Fusion Transcript Detection

EricScript framework (Version: 0.5.5b) is used for the discovery of fusion transcripts in paired end RNA-seq data (cite PMID: 23093608). Following steps are performed by the EricScript: (1) Mapping of the reads against the transcriptome (downloaded from the EricScript website, Homo sapiens, Ensembl version 73, Genome Build hg19/GRCh37), (2) Identification of discordant alignments and building of the exon junction reference, (3) Recalibration of the exon junction reference, (4) Scoring and filtering of the candidate gene fusions. The data is visualized using a custom webserver based on The JBrowse Genome Browser (PMID: 19570905). A simple perl script was written to filter through the list of candidate fusion transcripts and explored using the JBrowse Genome Browser (http://hegemon.ucsd.edu/RB-p1/fusion/fusion.php).The list was then manually filtered using BLAST on the NCBI website.

### cDNA Synthesis

cDNA was prepared from 400 ng of RNA using the High Capacity cDNA Reverse Transcription kit (Applied Biosystems), according to manufacturer's protocol.

### Quantitative RT-PCR

The expression of the specified target genes was quantified by q-RT-PCR, using Fast Start Universal SYBR Green Master with ROX (Roche), and was normalized to the expression of β-actin and normal samples. Custom designed primers were used.

### Y79 Transfection

2 x 10^5^ Y79 cells were seeded in a 24-well plate. Six wells each were transfected with pcDNA-DRAIC and pcDNA3-EmptyVector were transfected using Lipofectamine2000, according to manufacturer's protocol. The plasmids were a kind gift of Anindya Dutta (32).

### Cell Culture

The Y79 cell line was grown in RPMI medium supplemented with 20% FBS and 1X penicillin-streptomycin and cultured at 37°C, 5% CO_2_ incubator as per ATCC guidelines.

### Cell Growth Assay

Fifteen microlitre aliquot of cells are taken and diluted 1:1 with Trypan Blue solution. Cells are counted using a haemocytometer and the total number of cells are calculated.

### Data Access

Complete RNA-seq data from tumors and control retinal tissue uploaded to the NCBI GEO datasets (GSE125903).

## Author Contributions

KD extracted RNA from tumor and normal retinal samples. KD and SR generated cDNA and conducted RT-PCR from extracted RNA for second independent cohort analysis. RK tumor collection and data handling. PR and VK are the surgeons who obtained tumors. SK conducted the pathology on the tumor samples. KS, MB, and DS ran the bioinformatics analysis. LS conducted RNA fusion product analysis and MC conducted LNC-RNA correlation analysis. SE contributed to data handling and designed the experiments. WM designed experiments, helped with analysis, and wrote the manuscript.

### Conflict of Interest Statement

KS was employed by the company MedGenome. The remaining authors declare that the research was conducted in the absence of any commercial or financial relationships that could be construed as a potential conflict of interest.
