# Supplementary material for: Non-coding and Coding Transcriptional Profiles Are Significantly Altered in Pediatric Retinoblastoma Tumors
Source: Front Oncol. 2019 Apr 16;9:221. doi: 10.3389/fonc.2019.00221 (PMC6477087; doi:10.3389/fonc.2019.00221)
Supplement: Supplementary file 1 [file Presentation_1.pdf]

Patient ID	Sample No	Sample ID	Total Reads	Total data (GB)	Average read length (bp)	Avg. base quality (Phred)	Total data >= Q30 (%)	Total data >= Q20 (%)	GC (%)
C1	1	48428	160749358	16.07	100	35.08	92.31	95.50	49.84
1	2	48429	115547718	11.55	100	34.76	91.34	94.64	49.70
2	3	48430	89844222	8.98	100	34.61	90.75	94.15	45.75
3	5	48432	100707208	10.07	100	32.39	83.73	87.76	49.49
4	6	48433	105656294	10.57	100	32.86	83.31	89.20	47.29
5	7	48434	123753520	12.38	100	33.43	85.06	90.85	47.95
6	8	48435	130395444	13.04	100	33.28	84.40	90.45	46.89
C2	9	48436	137292256	13.73	100	33.11	83.90	90.00	49.45
7	10	48437	81481032	8.15	100	30.67	76.26	82.97	51.53

Supp. Table 1: Raw RNA-seq Data from retina and Retinoblastoma samples

Supp Table1

Patient ID	Sample No	Sample ID	Total Reads	Reads After Adapter Trimming	Reads After Contamination Removal	Aligned Reads
C1	1	48428	155890558	151413666	124041344	27372322
1	2	48429	109487988	98538684	44849696	53688988
2	3	48430	84750392	80783782	47515072	33268710
3	5	48432	76820448	72007794	32014848	39992946
4	6	48433	93770106	92569040	70279216	22289824
5	7	48434	114257030	113097440	86073066	27024374
6	8	48435	119992008	115365816	84183876	31181940
C2	9	48436	125180718	119513224	88776972	30736252
7	10	48437	57498540	57287434	587234	56700200

Supp. Table 2: Number of RNA-seq reads from retina and Retinoblastoma samples used for genome alignment

Supp Table 2

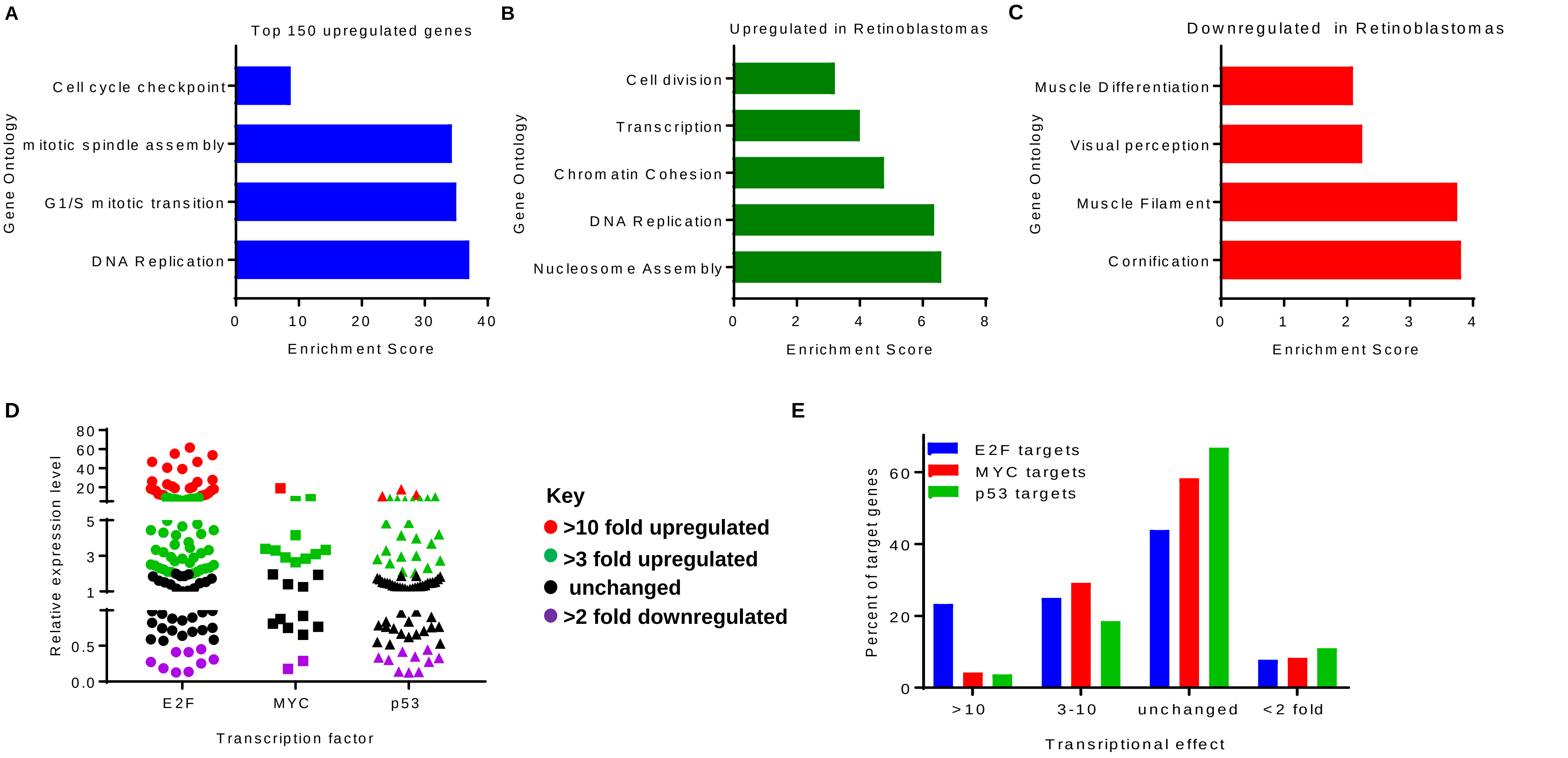

Supp. Figure 1: Functional classification of the coding gene changes in Retinoblastoma

(A) Gene Ontology of the most upregulated 150 coding genes in these tumors. **(B)** Gene Ontology of all of the upregulated coding mRNAs in Retinoblastoma. **(C)** Gene Ontology of all downregulated coding mRNAs in Retinoblastoma. **(D)** Relative expression levels of E2F, MYC and p53 target genes in Retinoblastoma. **(E)** Relative transcriptional effect on E2F, MYC and p53 target genes in tumors.

Rajasekaran _Supp Fig1

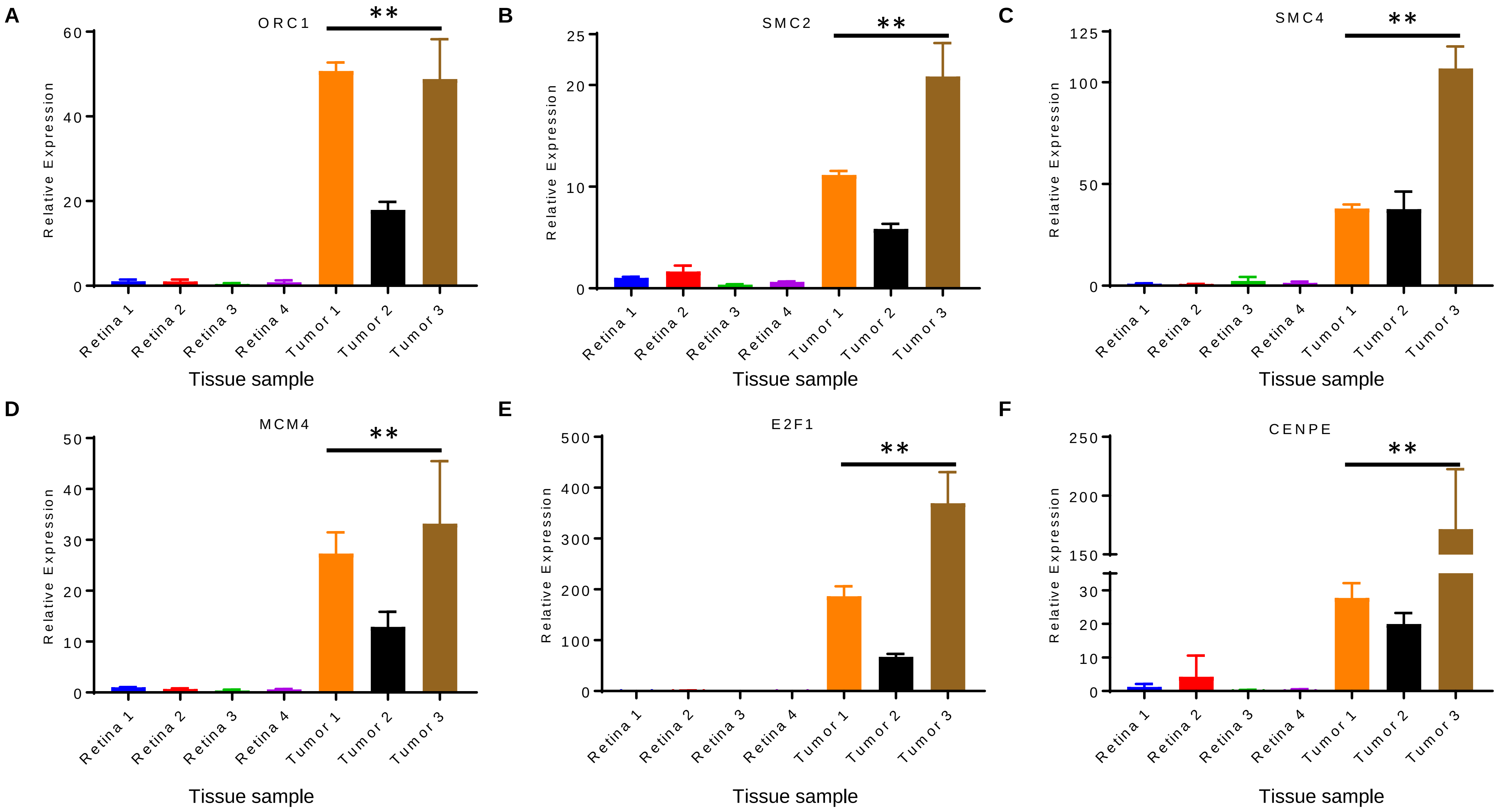

Supp. Figure 2: E2F target gene expression in an independent cohort of tumors

(A) RT-PCR Relative expression of ORC1 from four control retinas and three Retinoblastoma tumors from an independent cohort (*p<0.05). **(B)** RT-PCR Relative expression of SMC2, **(C)** SMC4, **(D)** MCM4, **(E)** E2F1 and **(F)** CENPE from control retina and three Retinoblastoma tumors from an independent cohort (*p<0.05).

Rajasekaran Supp Fig2

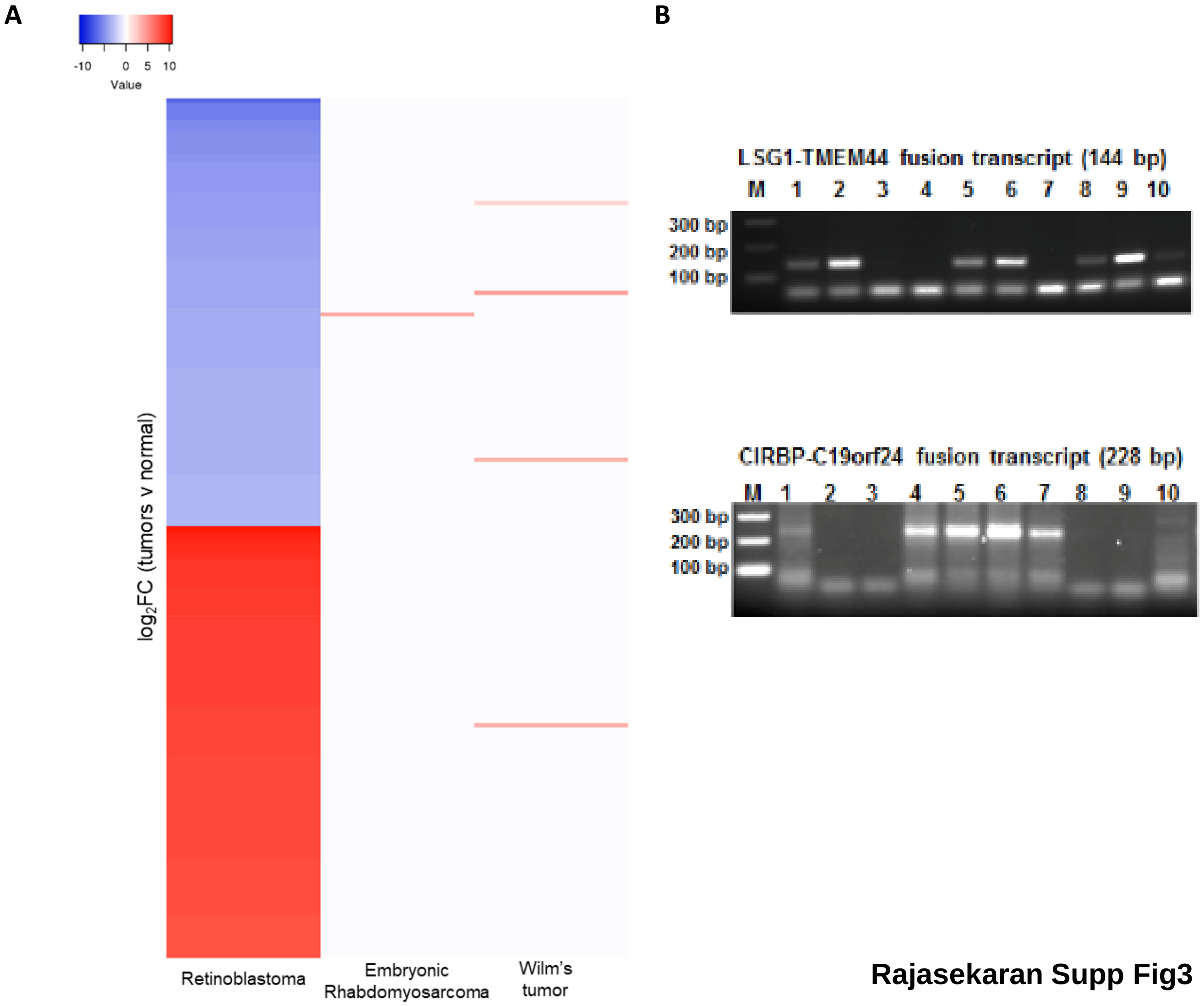

Rajasekaran Supp Fig3

Supp. Figure 3: Retinoblastoma RNA signature and RNA-fusions are unique.
(A) Heat map of upregulated (red) and downregulated (blue) genes in Retinoblastoma compared to other pediatric tumors. **(B)** RT-PCR results from RNA-fusion testing in Retinoblastoma tumors.
